# The role of rivaroxaban in eosinophilic myocarditis

**DOI:** 10.1093/ehjcr/ytac219

**Published:** 2022-05-26

**Authors:** Carla Bodagh, Chris Sawh, Pankaj Garg

**Affiliations:** Norwich Medical School, University of East Anglia, Norwich S10 2RX, UK; Cardiology, Norfolk and Norwich University Hospital NHS Trust, Norwich, UK; Norwich Medical School, University of East Anglia, Norwich S10 2RX, UK; Cardiology, Norfolk and Norwich University Hospital NHS Trust, Norwich, UK

**Keywords:** Eosinophilia, Myocarditis, Heart failure, Thrombosis, Rivaroxaban, Echocardiography, Magnetic resonance imaging, Factor Xa Inhibitors, Glucocorticoids

## Abstract

This is a case of eosinophilic myocarditis which had large left-ventricular thrombus seen on echocardiography and cardiac magnetic resonance imaging which was successfully treated with a highly selective direct Factor Xa inhibitor—rivaroxaban.

A 76-year-old gentleman with hypereosinophilia (6.21 × 10^9^/L, normal range 0.02–0.05) secondary to respiratory infection was referred to the cardiology team for shortness of breath. Trans-thoracic echocardiography demonstrated a dilated left ventricle (LV) and globally impaired systolic function and apical LV thrombus. Further examination by cardiac magnetic resonance imaging demonstrated an LV thrombus in the apex measuring 28 × 14 mm and sub-endocardial apical fibrosis on late gadolinium enhancement imaging. Endomyocardial pattern of fibrosis seen in the LV (‘V sign’) is typical for eosinophilic myocarditis. He was treated with Prednisolone 10 mg for 2 months and standard heart failure therapy. For the LV thrombus a highly selective direct Factor Xa inhibitor, rivaroxaban 20 mg was initiated instead of warfarin in light of logistic issues due to COVID-19 pandemic. After 9 months of treatment, the eosinophil count went down to 0.03 × 10^9^/L and the LV thrombus resolved to leave a fine rim of organized thrombus only, which has a relatively lower risk for systemic embolization (*Figure 1*).

**Figure 1:**
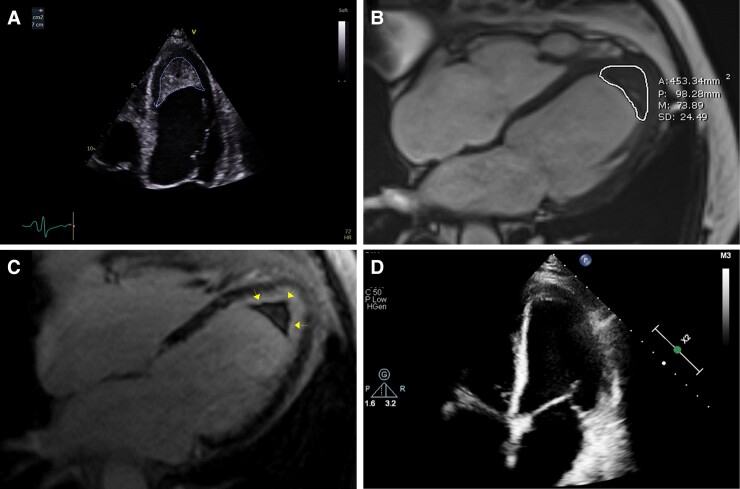
Pre-treatment: (*A*) B-mode echocardiography demonstrating apical LV mass. Left-ventricular ejection fraction is 33%, left-ventricular internal diameter end diastole 50 mm, and left-ventricular internal diameter end systole 41 mm. (*B*) Balanced steady-state precession cine MRI in four-chamber view demonstrating LV mass. (*C*) Late gadolinium enhancement confirms that it is homogenous hypointense mass in LV consistent with LV thrombus. Also, endomyocardial pattern of fibrosis of the left ventricle due to inflammation can be seen (yellow arrows). Post-treatment: (*D*) Repeat echocardiography after 9 months time confirms resolution of majority of the thrombus with now potentially less dangerous established transmural thrombus layer only. Left-ventricular ejection fraction is 37%, left-ventricular internal diameter end diastole 52 mm, and left-ventricular internal diameter end systole 44 mm.

This case highlights that a multi-faceted personalized treatment using disease-modifying agents and anticoagulation has a synergistic role in the resolution of myocardial inflammation and of the thrombus. This treatment strategy has been shown in limited cases previously.^1,2^ Even though we are not the first to report successful thrombus resolution with this treatment strategy, it is worth reporting our imaging findings to further add to the evidence database so that there is a motive for future prospective design studies, which explore the role of glucocorticoids and rivaroxaban in eosinophilic myocarditis.
